# Metabolic Changes of *Mycobacterium tuberculosis* during the Anti-Tuberculosis Therapy

**DOI:** 10.3390/pathogens9020131

**Published:** 2020-02-18

**Authors:** Julia Bespyatykh, Egor Shitikov, Dmitry Bespiatykh, Andrei Guliaev, Ksenia Klimina, Vladimir Veselovsky, Georgij Arapidi, Marine Dogonadze, Viacheslav Zhuravlev, Elena Ilina, Vadim Govorun

**Affiliations:** 1 Federal Research and Clinical Centre of Physical-Chemical Medicine, 119435 Moscow, Russia; d.bespiatykh@gmail.com (D.B.); andrewgull87@gmail.com (A.G.); ppp843@yandex.ru (K.K.); djdf26@gmail.com (V.V.); arapidi@gmail.com (G.A.); ilinaen@gmail.com (E.I.); govorun@hotmail.ru (V.G.); 2 Moscow Institute of Physics and Technology (State University), 141701 Dolgoprudny, Russia; 3 Research Institute of Phtisiopulmonology, 191036 St. Petersburg, Russia; marine-md@mail.ru (M.D.); jouravlev-slava@mail.ru (V.Z.)

**Keywords:** omics analysis, TB treatment, system analysis, Beijing B0/W148

## Abstract

Tuberculosis, caused by *Mycobacterium tuberculosis* complex bacteria, remains one of the most pressing health problems. Despite the general trend towards reduction of the disease incidence rate, the situation remains extremely tense due to the distribution of the resistant forms. Most often, these strains emerge through the intra-host microevolution of the pathogen during treatment failure. In the present study, the focus was on three serial clinical isolates of *Mycobacterium tuberculosis* Beijing B0/W148 cluster from one patient with pulmonary tuberculosis, to evaluate their changes in metabolism during anti-tuberculosis therapy. Using whole genome sequencing (WGS), 9 polymorphisms were determined, which occurred in a stepwise or transient manner during treatment and were linked to the resistance (GyrA D94A; *inhA* t-8a) or virulence. The effect of the *inhA* t-8a mutation was confirmed on both proteomic and transcriptomic levels. Additionally, the amount of RpsL protein, which is a target of anti-tuberculosis drugs, was reduced. At the systemic level, profound changes in metabolism, linked to the evolution of the pathogen in the host and the effects of therapy, were documented. An overabundance of the FAS-II system proteins (HtdX, HtdY) and expression changes in the virulence factors have been observed at the RNA and protein levels.

## 1. Introduction

*Mycobacterium tuberculosis* bacteria are among the deadliest microbial pathogens in the world. Over 10 million new cases and 1.3 million lethal cases are registered annually [1]. Despite the reduction in the total tuberculosis (TB) incidence rate, the situation remains extremely tense due to the distribution of resistant forms. Among them, the multidrug-resistant- (MDR, resistance to at least isoniazid (INH) and rifampicin (RIF)) and extensively drug-resistant- (XDR, MDR phenotype with additional resistance to any fluoroquinolone (FQ) and at least one of the second-line injectable drugs capreomycin (CAP), kanamycin (KAN) or amikacin (AM)) tuberculosis pose a serious challenge for global TB control and makes successful treatment difficult or impossible [2].

Directly observed treatment (DOT) and DOT plus are the current standards of treatment strategies for the drug-susceptible and resistant forms of tuberculosis, respectively [3]. Although these protocols show high efficiency, the emergence and fixation of resistance to new antibiotics have been observed all over the world [4,5,6]. There are many reasons for this and not all of them have been fully elucidated, but it is worth mentioning that pathogen resistance develops in the patient, as the bacterium is a human obligate parasite. Treatment-driven positive selection leads to: the emergence of resistant clones within a population, their growth in a heteroresistant state and further ultimate fixation of a drug-resistant variant [7].

In recent years, multi-omics technologies have been increasingly used to understand the processes taking place in bacterial cells during the formation of resistance. For instance, the whole genome sequencing allows not only to undoubtedly distinguish mixed and unmixed infections in the patient, but also to define heterogeneity within one population [8,9,10]. The latter is often associated with the acquisition of certain drug resistance-conferring mutations, which occur independently in multiple clones and have different fitness costs, and, as a consequence, different probabilities of fixation. Apart from this, novel genetic variants may be fixed in the population by the mechanism of hitchhiking or independently [10]. A comprehensive review of these mutations has been recently published and demonstrated that most of these variants occurred in the genes, associated with the compensatory mechanisms, virulence, or relapse and survival, but their consequences remain unclear [11]. 

At the same time, commonly revealed mutations do not reflect all changes occurring in the cell [12]. Recently, a total of 139 genes were shown to be differentially expressed among serial isolates, while only 15 mutations were different between the strains [10]. In another work, the abundance of 46 proteins differed between resistant and sensitive strains. [13]. The *in vitro* experiments studying the effect of anti-tuberculosis drugs on bacteria have shown changes in the abundance of proteins related to amino acid, carbohydrate, and nucleotide metabolism [14]. 

In the present study, for the first time the author provided multi-omics analysis of three consecutive *M. tuberculosis* isolates from the same patient, to evaluate the changes in bacterial metabolism during anti-tuberculosis therapy. Based on whole genome sequencing (WGS) data, a stepwise accumulation of polymorphisms, explaining the occurrence of phenotypic resistance to fluoroquinolones and high concentrations of isoniazid, was determined. Moreover, at the proteomic and transcriptomic levels, changes were found in the loci associated with drug-resistance and virulence that only partly can be explained by mutations in the corresponding gene regions. 

## 2. Results and Discussion

### 2.1. Clinical Isolates Characteristic

Three isolates from the patient with pulmonary tuberculosis have been subjected to multi-omics analysis. The first sample, RUS_B0, was isolated in 2008 before receiving TB chemotherapy. The second one, isolate 3955, was obtained one year after the treatment with high-doses of isoniazid, para-aminosalicylic acid (PASK), capreomycin, cycloserine, levofloxacin, pyrazinamide, azithromycin, clarithromycin, and amoxicillin with clavulanic acid. The 2093 sample was isolated after three years of treatment (surgery and chemotherapy according to the individual regimen).

Drug-susceptibility testing (DST) analysis has shown that the RUS_B0 isolate was MDR, while both 3955 and 2093 isolates were XDR. Phenotypic resistance to FQ evolved in the 3955 isolate after a year of treatment and was retained in the isolate 2093. Additionally, the 2093 isolate showed resistance to higher doses of isoniazid (10 mg/L).

Based on genotyping analysis, strains under study belonged to the Beijing B0/W148 cluster. Both IS*6110* RFLP and 24-loci MIRU-VNTR typing have not revealed any changes in 3955 and 2093 strains relative to RUS_B0. 

### 2.2. Intra-host Genome Evolution of M. tuberculosis Serial Isolates 

**Figure 1:**
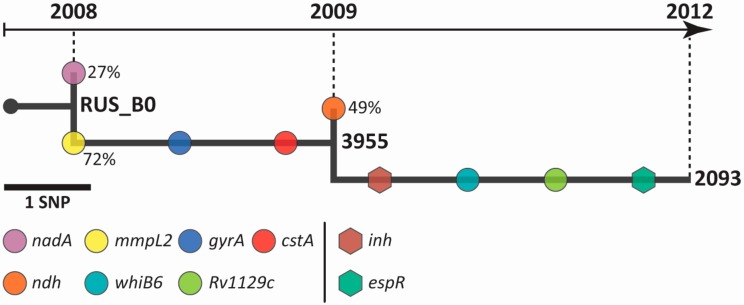
***M. tuberculosis* genome evolution within the patient.** Sample collection time points are indicated by the numbers above the arrow. Circles and hexagons represent mutations in genes and promoter regions, respectively. Variant allele frequency is shown by the numbers beside circles.

Three isolates of *M. tuberculosis* from a single patient, collected during different time points of treatment, were sequenced at a median depth of 383x coverage. Allele frequency of > 25% was taken as a threshold for sub-populations differentiation. In comparison to H37Rv, nine single nucleotide polymorphisms (SNPs) were variable among selected serial isolates, and seven of them were fixed in the last sample (Figure 1, Table S1).

The initial isolate RUS_B0 consisted of two populations carrying specific polymorphisms. The dominant population (72% of reads) carried synonymous SNP in the *mmpL2* gene (pos. 598098c-t; I300I), while the minor population had a substitution P287S in the NadA protein (pos. 1795614c-t). 

On the second time point (12 weeks, isolate 3955), the predominant population has been ultimately fixed (all reads had a mutation in *mmpl2*) in the sample, as well as additional polymorphisms appeared: 3427440g-t (P66P) and 7582a-c (D94A) in the *cstA* and *gyrA* gene, respectively. As in the first case, the isolate 3955 consisted of two populations. One of them (49% of reads) had a 2102714g-a mutation in the *ndh* gene, leading to the T110I substitution. Of note, another substitution in this codon, T110A, was previously described as associated with isoniazid resistance, but it was found only in addition to the *ahpC* mutation [15]. Although the presence of this mutation did not affect the isoniazid resistance of the 3955 isolate, it was assumed that it was caused by the high-doses of INH used during the treatment.

It was also observed that the sub-population with a mutation in the *ndh* gene has not been fixed in the patient and in the third time point, isolate 2093, all reads corresponded to the reference. Nevertheless, a novel mutation (*inhA* t-8a), associated with resistance to INH, has emerged in this isolate. In addition to the drug resistance-associated mutations, isolate 2093 carried SNPs in *Rv1129c* (1253465c-t; S357N) and *whiB6* (4338344a-g; W60R) genes, as well as in the promoter region of the *espR* gene (c-90t).

### 2.3. Analysis of Drug Resistance Genes on Multi-omics Level

To evaluate the changes in bacterial metabolism during anti-tuberculosis therapy, multi-omics analysis of the serial strains was performed. The transcriptomic analysis resulted in the identification and quantification of 3889, 3894, and 3855 transcripts (TPM ≥ 1) in the RUS_B0, 3955, and 2093 strains, respectively (Table S2). According to the proteome analysis, 1941, 1728, and 1989 proteins were identified for the RUS_B0, 3955, and 2093 strains, respectively (Table S3). For quantitative proteomic analysis, 1358 proteins identified in all three strains were used. Over- and underrepresented proteins for each pair of strains are presented in Table S4. 

According to DST results, all strains had the same following drug resistance-associated mutations: STR (RpsL: K43R), INH (KatG: S315T), RIF (RpoB: S450L), EMB (EmbB: Q497R), ETH (*ethA*: 110delA), KAN, CAP, AM (*rrs*: a1401g), PZA (*pncA*: t-11c) (Table 1). On the transcriptomic and proteomic levels, differences in the *rpoB*, *pncA, embB* genes and related proteins, as well as in the *rrs* gene, were not observed between the strains. Proteomic analysis revealed that in 2093 samples, abundance of RpsL and seven other ribosome proteins (Rv0055, Rv0714, Rv0720, Rv0722, Rv0979A, Rv1298, and Rv2412) was lower compared to the parental strains. This finding may be related to the fact that ribosomal proteins can be targeted by anti-tuberculosis drugs [16], and, particularly, by pyrazinamide, which was used for the treatment. 

For the ETH resistance-associated gene *ethA* carrying a frameshift mutation in the coding sequence, a reduction in gene expression during the treatment was found. Previously, the author has shown an increased level of *ethA* transcripts in the RUS_B0 (almost eight times) compared to the H37Rv. Its open reading frame contains numerous frame-shifts and stop codons precluding protein synthesis. The author hypothesized that MymA (Rv3083) can partly substitute the function of the inactivated gene [17]. In the present study, an up-regulation of *mymA* gene in 3955 strain compared to RUS_B0 was detected.

Starting from the second time point, the studied isolate 3955 acquired a D94A substitution in GyrA, which was also found in the 2093 isolate (Figure 1, Table 1). This mutation linked with resistance to FQ and, thus, correlates with the DST results. According to the RNA-seq and proteomics data, the levels of the *gyrA* transcript and correspondent protein were the same in all the strains. However, another gyrase subunit, GyrB, was overrepresented in the 2093 isolate.

The isoniazid-resistant strains carrying the S315T substitution in the KatG have acquired an additional mutation in the promoter region of the *inhA* gene (isolate 2093) (Figure 1, Table 1). Recently, it was shown that combination of KatG S315T and *inhA* c-15t is associated with high-level resistance (MIC ≥ 19.2 mg/L) to INH [18]. According to our study, the isolate 2093, harboring KatG S315T and *inhA* t-8a, was also resistant to the high drug concentrations (MIC=10 mg/L). In turn, the whole transcriptome analysis of the *mabA – inhA* operon revealed an increased level (7-fold) of corresponding gene transcripts in the 2093 isolate compared to the RUS_B0. It is known that INH resistance is associated with hyperproduction of the target InhA protein [19]. However, at the proteomic level, only an overabundance of MabA protein was found (changes in InhA were not detected). As mentioned above, the 2093 strain was resistant to higher concentrations of isoniazid than the RUS_B0 and 3955. InhA and MabA are both functionally and structurally related. Thus, MabA activity is also inhibited by isoniazid [20]. All of this may be evidence of high resistance of the bacterial cells.

Previously, significant differences in the levels of the *katG* and *hspX* transcripts in the INH-resistant and susceptible strains have been shown [13]. According to our data, the level of *katG* transcript was the same, while the expression of the *hspX* was higher in the 2093 and 3955 strains, compared to the RUS_B0. The abundance of KatG protein was the same after one year of therapy, but increased in the 2093 strain, which is consistent with the data, previously obtained for the INH-resistant Beijing isolates [13]. Regarding the HspX protein, its increased representation in the 3955 strain compared to RUS_B0, which went down in the strain 2093, was observed. Previously, it was proposed that HspX can be used as a marker of the disease, including its latent form [21,22]. A reduced representation of this protein in the resistant strain casts doubts on its usefulness. 

### 2.4. Non-Specific Bacterial Response to Anti-tuberculosis Therapy 

A number of studies have shown that mutations, acquired during treatment, do not reflect all metabolic changes occurring in the cell [10,12]. In our study, an increased level of the Rv2971 protein in the 2093 strain compared to the RUS_B0 and 3955 was determined. This protein is an oxidoreductase that is necessary for the growth of mycobacteria and has a potential role in detoxification of toxic metabolites. Previous studies have shown that this enzyme was differentially represented between INHr and INHs strains [23,24] and for STR [25]. In the present study, higher transcript levels of *Rv2971* in the 2093, compared to other strains, were detected. Taken together, these may reflect the strain’s resistance to high-doses of isoniazid.

Previously, the author documented an increased abundance of proteins involved in lipid metabolism, in particular, those responsible for the biosynthesis of long-chain fatty acids, in strains recovered after anti-TB treatment [26]. Another study has identified differences in the abundance of Rv0241 (HtdX) and Rv3389 (HtdY) that belongs to the FAS-II system, and Rv0242c (FabG4) related to FAS-I in the INH-resistant and susceptible strains [13]. Here, the increased representation of both FAS-II system proteins in the 2093 strain, as compared to the RUS_B0, is demonstrated. However, no changes in the abundance of the FabG4 fatty acid synthase were detected. Increased representation of the Rv0469 (UmaA) protein, which is responsible for the synthesis of mycolic acids, may facilitate the formation of the cell membrane. This can prevent penetration of the anti-TB drugs, lowering their intracellular concentration and, thus, establishing favorable conditions for the survival of the pathogen and subsequent development of drug resistance. It has been frequently suggested that enzymes of the lipid metabolism play an important role in the positive selection of mycobacteria [11], and fatty acids can be considered as virulence factors of these pathogens [17,26]. 

### 2.5. Variability in the Virulence Factors Representation 

**Figure 2:**
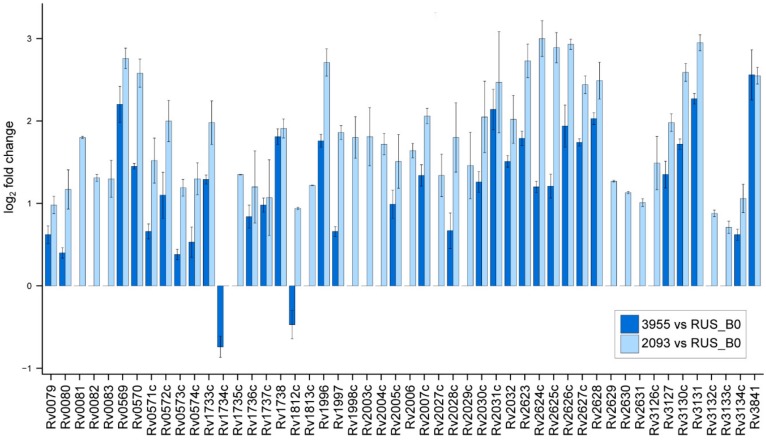
**DosR regulon expression.** Bars indicate fold changes in gene expression in 3955 (blue bars) and 2093 (light blue bars) strains compared to the RUS_B0 strain. Error bars depict standard error of the mean from three independent experiments.

It is well known that the DosR system is involved in the response of mycobacteria to the action of the host cell immune system and to the changing environmental conditions [27,28]. The author has previously shown that under normal growth conditions the DosR regulon proteins were poorly represented in the Beijing B0/W148 cluster strains compared to the H37Rv. This is correlated with an increased abundance of the same proteins and corresponding transcripts in cluster strains under stress conditions [17]. In the present study, a statistically significant increase in the transcription levels of the DosR regulon genes on the background of anti-tuberculosis therapy was observed (Figure 2). The level of all DosR regulon genes has increased in the 3955 and 2093 compared to the RUS_B0 strain, except for the Rv1734c and Rv1812c genes, of which transcription levels were lower in the 3955 strain. 

A mutation in the promoter region of *espR* was only detected in the 2093 strain. It has led to a decrease in the protein abundance and transcript level (albeit, not statistically significant). The virulence phenotype of bacteria is often implemented through protein hyperproduction *in vivo* [29], which is mediated by some intermediate activators. This also explains the low representation of the EspR and EspC proteins in the analyzed strain.

In the present study, a low abundance of the PtpA protein after the anti-TB therapy in the 2093 strain was observed. According to the transcriptome analysis, expression of the *ptpA* was higher in the 2093 strain. Concordantly, representation of the bifunctional protein Rv2228c, which is associated with PtpA, was higher. At the same time, the level of the *Rv2228c* transcript was higher in the 3955 strain, compared to the RUS_B0. It is known that PtpA (Rv2234) and PtpB (Rv0153c) are secreted proteins, which play an important role in the pathogen interaction with the host cell [30] and have orthologs among other pathogenic pro- and eukaryotes [31]. Rv2228c is presumably involved in bacterial replication, as it is only authenticated RNase HI in *M. tuberculosis*.

## 3. Materials and Methods

### 3.1. Mycobacterium Tuberculosis Strains and Growth Conditions

Three strains (RUS_B0, 3955, 2093) of *Mycobacterium tuberculosis* Beijing B0/W148 cluster from the same patient were used. Strains were grown in liquid Middlebrook 7H9 medium with Oleic Albumin Dextrose Catalase (OADC) supplement (Becton Dickinson, Franklin Lakes, USA). The cultures (90 mL) were grown in three biological replicates per strain in a cell culture flask (T175, Eppendorf, Germany) in a horizontal position. *M. tuberculosis* strains were incubated at 37°C for 12 days with constant shaking (5 rpm) until OD_600_ was reached to 0.4 [32]. The bacterial suspension from each flask was immediately aliquoted in a sterile 50 mL (for transcriptome and proteome analysis) and 15 mL (for WGS) tubes at room temperature (RT).

For WGS, an aliquot (10 mL) was centrifuged at 3200g for 10 min (RT) and the pellet was stored at -20 °C. Samples (40 mL) for transcriptomic analysis were centrifuged at 3200g for 10 min (RT) and cells pellet was frozen in liquid nitrogen and stored at −80 °C until use. For proteomic analysis, cells (40 mL of culture) were harvested by centrifugation at 3500g, 4°C for 5 min and washed with Tris-HCl and 2% Triton-X100 (pH 7.5–8). Cells were precipitated by centrifugation at 4500g, 4°C for 15 min. The pellet was frozen in liquid nitrogen and stored at −80 °C until required.

The susceptibility testing was done using the BACTEC MGIT 960 Culture system (Becton Dickinson) following the manufacturer’s protocol.

### 3.2. Genomic Analysis

Genomic DNA was isolated from the strains using standard extraction methods [33]. To verify that the strains belong to a Beijing B0/W148 cluster, the PCR assay was performed as described previously [34]. Spoligotyping, IS*6110*-RFLP and 24-MIRU-VNTR typing were performed as described in [35], [33], and [36], respectively.

Whole genome sequencing of the strains was performed on Illumina HiSeq 2500 Sequencing Platform according to the manufacturer’s instructions. Raw data were deposited in the NCBI Sequence Read Archive under accession number PRJNA421323. The circular genome of RUS_B0 strains was assembled and deposited in the NCBI earlier (CP030093.1) [37]. WGS reads were aligned to the *M. tuberculosis* H37Rv (NC_000962.3) and RUS_B0 (CP030093.1) genome sequences using Bowtie 2 [38]. SAMtools (v.0.1.18) [39], FreeBayes (v.1.1.0) [40] and Snippy (v.4.3.6) [41] were used for variant calling [39,42]. For FreeBayes and Snippy SNPs with a minimum mapping quality of 20, minimum coverage of 10 and alternate fraction of 0.9 were taken.

A comprehensive list of drug-resistance mutations to first- and second-line drugs were used to determine genetically resistant strains [43]. The identification of IS*6110* integration sites was carried out using ISMapper pipeline [44].

### 3.3. Transcriptomic Analysis

#### 3.3.1. RNA Extraction

Total RNA was isolated from all *M. tuberculosis* cultures as previously described [32]. In brief, bacteria were re-suspended in 1 mL Trizol (Invitrogen) and added to a 2-mL Lysing Matrix B (MP Bio, USA). Cells were disrupted by bead-beating twice for 1 min with a 2-min interval on ice. The suspension was then transferred to a new tube, where chloroform extraction was performed. RNA was precipitated by adding 0.7 volume of isopropanol and washed with 70% ethanol, air-dried and re-suspended in 100 µL DEPC-treated water. 

DNase treatment was carried out with TURBO DNA-free kit (Thermo Fisher Scientific) in volumes of 100 µL and further with the RNase-Free DNase Set (Qiagen, Germany) according to the manufacturer’s protocol. RNA cleanup was performed with the RNeasy Mini Kit (Qiagen) according to the RNA Cleanup protocol and stored at −70°C until further use. DNA contamination was evaluated by PCR, using primers for amplification of the IS*6110* fragment (PolyTub, Lytech, Russia). The concentration and quality of the total extracted RNA were checked by using the Quant-it RiboGreen RNA assay (Thermo Fisher Scientific) and the RNA 6000 pico chip (Agilent Technologies), respectively.

#### 3.3.2. RNA-seq and Analysis

Total RNA (1 - 2.5 µg) was used for library preparation. Ribosomal RNA was removed from the total RNA and libraries were prepared using the ScriptSeq Complete kit (Epicentre/Illumina, Madison, USA), according to the manufacturer’s protocol. Subsequently, RNA cleanup was performed with the Agencourt RNA Clean XP kit (Beckman Coulter, Brea, USA). The library underwent a final cleanup using the Agencourt AMPure XP system (Beckman Coulter) after which the libraries’ size distribution and quality were assessed using a high sensitivity DNA chip (Agilent Technologies). Libraries were subsequently quantified by Quant-iT DNA Assay Kit, High Sensitivity (Thermo Fisher Scientific). Finally, equimolar quantities of all libraries (12 pM) were sequenced by a high throughput run on the Illumina HiSeq using 2×125 bp paired-end reads and a 5% Phix spike-in control. Before loading the cBot system, the libraries were incubated at 98°C for 2 minutes and then cooled on ice to improve the hybridization of the GC-rich sequences. In total, 114 and 110 million paired reads were obtained corresponding to 14 and 13 billion nucleotide bases for H37Rv and RUS_B0, respectively. The dataset of RNA-Seq analysis was deposited to the NCBI with the project name PRJNA421323.

Adaptors were trimmed with the Trimmomatic v0.33 tool [45]. Quality control on raw reads was carried out with FASTQC v0.11.5 [46]. The Kallisto v0.46.0 [47] software was used for the reads mapping and abundance estimation. Differential expression analysis was performed using edgeR v3.26.8 [48] package, integrated in the Degust v4.1.1 [49] web-tool. Only genes with count per million (CPM) ≥ 1 were analyzed further. Genes were filtered based on false discovery rate cutoff (FDR) ≤ 0.05 and minimum expression fold change (FC) ≥ 2. The plots were generated using the ggplot2 package in R.

### 3.4. Proteomic Analysis

#### 3.4.1. Protein Extraction and Trypsin Digestion

The bacterial pellet was lysed and protein was extracted as described previously [17]. Protein concentration was measured by the Bradford method [50] using the Bradford Protein Assay Kit (Bio-Rad, Hercules, USA).

Proteolytic in-gel digestion was performed in three biological and two technical replicates: 1) sample, fractionated into 6 parts; 2) total load sample as described previously [51]. Peptides were cleaned using C18 Sep-Pak columns (Waters, Milford, USA) [17].

#### 3.4.2. LC-MS/MS Analysis

Peptides were separated with high-performance liquid chromatography (HPLC, Ultimate 3000 Nano LC System, Thermo Fisher Scientific,) in a 15-cm long C18 column with a diameter of 75 μm (Acclaim® PepMap™ RSLC, Thermo Fisher Scientific). The peptides were eluted with a gradient from 5% to 35% of buffer B (80% acetonitrile, 0.1% formic acid) over 115 min at a flow rate of 0.3 μl/min. Total run time including 15 min to reach 99% buffer B, flushing 10 min with 99% buffer B and 15 min re-equilibration to buffer A (0.1% formic acid) amounted to 65 min. Further analysis was performed with a Q Exactive HF mass spectrometer (Q ExactiveTM HF Hybrid Quadrupole-OrbitrapTM Mass spectrometer, Thermo Fisher Scientific). Mass spectra were acquired at a resolution of 60,000 (MS) and 15,000 (MS/MS) in a range of 400-1500 m/z (MS) and 200-2000 m/z (MS/MS). An isolation threshold of 67,000 was determined for precursor selection, and up to the top 25 precursors were chosen for fragmentation with high-energy collisional dissociation (HCD) at 25 V and 100 ms activation time. Precursors with a charged state of +1 were rejected and all measured precursors were excluded from measurement for 20 s. From 2 to 4 technical runs were analyzed for each sample.

#### 3.4.3. Protein Identification and Quantitation

Raw data was captured from the mass spectrometer and converted to MGF (Mascot Generic Format) files using ProteoWizard with the following parameters: peakPicking true 2, msLevel 2, zeroSamples removeExtra [52]. For thorough protein identification, the generated peak lists were searched with the MASCOT (v 2.5.1, Matrix Science Ltd, UK) and X! Tandem (VENGEANCE, 2015.12.15, The Global Proteome Machine Organization) search engines. Database-searching parameters were as follows: tryptic hydrolysis, no more than one missed site, the precursor and fragment mass tolerance were set at 20 ppm and 50 ppm, respectively. Oxidation of methionine was set as a possible modification, carbamidomethylation of cysteine as a fixed. For X! Tandem, parameters that allowed a quick check for protein N-terminal residue acetylation, peptide N-terminal glutamine ammonia loss or peptide N-terminal glutamic acid water loss were selected. Resulting files were submitted to the Scaffold 4 software (v 4.2.1, Proteome Software, Inc, USA) for validation and further analysis. For protein identification, the proteomic databases for the RUS_B0 (RefSeq: CP030093.1) and reference strain H37Rv (RefSeq: NC_000962.3) genomes were used. Additionally, in proteome searching database peptides containing strain-specific single amino acid polymorphisms were added according to the approach described earlier [51]. The local false discovery rate scoring algorithm with standard experiment-wide protein grouping was used. A 1% FDR threshold was applied to search results from individual datasets. For all detected proteins, functional categories (TubercuList v 2.6 (http://tuberculist.epfl.ch/)) and subcellular localizations (PSORTdb v 3.0 (http://db.psort.org/)) were established.

For label-free quantitation, raw MS data files (.wiff files) were imported and processed in Progenesis LC-MS software v.4.1 (Nonlinear Dynamics, Newcastle, UK). The results of peptide quantitation were normalized using an iterative median-based normalization as implemented in the Progenesis software. Differences in the abundance of a protein between the three biological replicates of all strains were evaluated using a two-sided unpaired Student’s T-test. *P*-values < 0.05 were considered statistically significant. Adjusted *p*-values for multiple tests (*q*-values) were generated using the Benjamini–Hochberg method [53].

## 4. Conclusions

This is a first report of the system omics analysis of *M. tuberculosis* Beijing B0/W148 cluster strains isolated from one patient at different stages of TB therapy. A clear correlation between genetic and phenotypic resistance to the antibiotics was demonstrated, documenting changes in the lipid metabolism, and production of virulence factors on the proteomic and transcriptomic levels. These changes imply that the anti-tuberculosis therapy play a crucial role in the regulation of bacterial biochemical pathways.

We show that integrating various approaches can advance our knowledge of the role and significance of microevolution in *M. tuberculosis* infection.

## Figures and Tables

**Table 1:** Results of phenotypic and genetic drug susceptibility for serial strains.

Drug* (Resistance Associated Gene/Protein)	Clinical Isolates		
	RUS_B0	3955	2093
STR (Rpsl)	R (K43R)	R (K43R)	R (K43R)
INH (KatG) (*inhA*)	R (S315T)	R (S315T)	R (S315T) R+** (t-8a)
RIF (RpoB)	R (S450L)	R (S450L)	R (S450L)
EMB (EmbB)	R (Q497R)	R (Q497R)	R (Q497R)
ETH (*ethA*)	R (110delA)	R (110delA)	R (110delA)
FQ (GyrA)	S	R (D94A)	R (D94A)
KAN, CAP, AM (*rrs*)	R (a1401g)	R (a1401g)	R (a1401g)
PZA (*pncA*)	R (t-11c)	R (t-11c)	R (t-11c)

*STR - streptomycin, INH - isoniazid, RIF - rifampicin, EMB - ethambutol, ETH - ethionamide, FQ - fluoroquinolone, KAN - kanamycin, CAP - capreomycin, AM- amikacin, PZA - pyrazinamide; R - resistant, S - susceptible. **high-dose resistance (MIC=10mg/L).

## Supplementary Materials

The following are available online at https://www.mdpi.com/2076-0817/9/2/131/s1. Table S1: Variable SNPs among tree clinical isolates. Table S2: List of identified and quantified transcripts for RUS_B0, 3955 and 2093 strains. Table S3: Proteins and peptides identified in RUS_B0, 3955 and 2093 strains. Table S4: Quantitative proteomic analysis of RUS_B0, 3955 and 2093 strains.
